# Impact of Irrigation Strategies on Tomato Root Distribution and Rhizosphere Processes in an Organic System

**DOI:** 10.3389/fpls.2020.00360

**Published:** 2020-03-27

**Authors:** Meng Li, Jennifer E. Schmidt, Deirdre G. LaHue, Patricia Lazicki, Angela Kent, Megan B. Machmuller, Kate M. Scow, Amélie C. M. Gaudin

**Affiliations:** ^1^Department of Plant Sciences, University of California, Davis, Davis, CA, United States; ^2^Department of Crop and Soil Sciences, Washington State University, Mount Vernon, WA, United States; ^3^Department of Land, Air, and Water Resources, University of California, Davis, Davis, CA, United States; ^4^Department of Natural Resources and Environmental Science, University of Illinois at Urbana-Champaign, Urbana, IL, United States; ^5^Natural Resource Ecology Laboratory, Colorado State University, Fort Collins, CO, United States; ^6^Department of Soil and Crop Science, Colorado State University, Fort Collins, CO, United States

**Keywords:** root distribution, rhizosphere, organic system, soil enzyme activity, N-cycling functional genes, mycorrhizae, subsurface drip irrigation

## Abstract

Root exploitation of soil heterogeneity and microbially mediated rhizosphere nutrient transformations play critical roles in plant resource uptake. However, how these processes change under water-saving irrigation technologies remains unclear, especially for organic systems where crops rely on soil ecological processes for plant nutrition and productivity. We conducted a field experiment and examined how water-saving subsurface drip irrigation (SDI) and concentrated organic fertilizer application altered root traits and rhizosphere processes compared to traditional furrow irrigation (FI) in an organic tomato system. We measured root distribution and morphology, the activities of C-, N-, and P-cycling enzymes in the rhizosphere, the abundance of rhizosphere microbial N-cycling genes, and root mycorrhizal colonization rate under two irrigation strategies. Tomato plants produced shorter and finer root systems with higher densities of roots around the drip line, lower activities of soil C-degrading enzymes, and shifts in the abundance of microbial N-cycling genes and mycorrhizal colonization rates in the rhizosphere of SDI plants compared to FI. SDI led to 66.4% higher irrigation water productivity than FI, but it also led to excessive vegetative growth and 28.3% lower tomato yield than FI. Our results suggest that roots and root-microbe interactions have a high potential for coordinated adaptation to water and nutrient spatial patterns to facilitate resource uptake under SDI. However, mismatches between plant needs and resource availability remain, highlighting the importance of assessing temporal dynamics of root–soil–microbe interactions to maximize their resource-mining potential for innovative irrigation systems.

## Introduction

Plasticity in root exploitation of soil resource heterogeneity and microbially mediated nutrient cycling processes in the rhizosphere provide the foundation for plant adaptation, productivity, and resource use efficiency (Philippot et al., 2013). In agroecosystems, irrigation practices are designed to deliver water to crop roots, but the spatiotemporal dynamics of resource availability are rarely perfectly coupled with plant demand (Howell, 2001). This mismatch arises from limited research on how roots and rhizosphere processes respond to water and nutrient dynamics that are shaped by irrigation methods over space and time (Raine et al., 2007). The neglect of root and rhizosphere interactions during the implementation of new irrigation practices, especially when management changes are made based on experience or external reasons, can lead to inefficient use of costly inputs and damaging losses into the environment (Vázquez et al., 2006; Thompson et al., 2007). Filling in this knowledge gap is critical to decreasing the environmental footprint of agriculture, improving water and nutrient use efficiency, and maximizing benefits from novel water-saving irrigation technologies (Dodd, 2009).

Plants can regulate root system development to maximize acquisition of soil moisture and nutrient resources whose distribution is affected by irrigation (Jin et al., 2017; Schmidt and Gaudin, 2017). This plasticity can be achieved through different strategies including spatially targeted root proliferation to mine nutrient- and moisture-rich patches while avoiding resource-poor areas (Hodge, 2009). Plasticity occurs over time as well: plants can develop rapid root regrowth along with increased root physiological activity, such as N uptake and hydraulic conductivity, following the soil rewetting caused by rainfall or irrigation events (BassiriRad and Caldwell, 1992; Huang and Eissenstat, 2000). A better understanding of root responses to wetting patterns caused by different irrigation strategies will contribute to the design of new resource-saving irrigation technologies and the development of effective root ideotypes for irrigated landscapes (Schmidt and Gaudin, 2017).

Integrating rhizosphere processes mediated by root-associated microbes with root developmental patterns is also necessary to enhance our mechanistic understanding of plant adaptation to resource availability under different irrigation strategies (Schmidt and Gaudin, 2017). Soil microorganisms participate in vital biogeochemical processes that influence plant nutrient uptake and nutrient retention (Bardgett et al., 2014). For instance, nutrient-limited bacteria and fungi release extracellular enzymes that regulate the depolymerization of soil organic matter (SOM) and thus mediate the overall carbon (C) and nitrogen (N) cycling rate in soils (Frossard et al., 2000; Schimel and Bennett, 2004). In addition, soil microorganisms compete with roots for mineralized soil nutrients such as inorganic N (Jackson et al., 2012; Coskun et al., 2017). Quantifying microbial genes involved in N cycling pathways in the rhizosphere, including nitrification (*amoA*) and denitrification pathways (*nirS, nirK, nosZ*), can indicate N transformation rates and thus N availability to plants (Jackson et al., 2008; Morales et al., 2010). Plants also benefit from direct associations with arbuscular mycorrhizal (AM) fungi for the uptake of water and low mobile nutrients, especially phosphorus (P), through extended mycorrhizal hyphae networks in soils (Augé, 2001; Treseder, 2004). These microbial processes can be accelerated or inhibited by different soil moisture patterns and wet-dry cycles induced by irrigation, thus regulating plant nutrient uptake and soil nutrient cycling (Fierer and Schimel, 2002; Burger et al., 2005). However, in previous studies, responses of rhizosphere microbial processes to irrigation have attracted limited attention and are often considered separately from root developmental traits (Zotarelli et al., 2009b; Wang et al., 2018). This limits the mechanistic understanding of how root–soil–microbe interact and adapt to resource availability under different irrigation strategies.

A better understanding of responses of root-soil-microbe interactions to spatial patterns of resource availability has critical implications for agricultural management optimization, especially for organically managed systems. Plants grown under organic management rely on microbe-mediated processes along with the nutrient mining of roots to maintain adequate levels of plant nutrition and productivity (Jackson et al., 2012). However, in organic systems, root developmental and rhizosphere responses and their relationship to plant productivity are difficult to predict, as resource availability varies rapidly with microbially mediated mineralization-immobilization dynamics and the outcome of plant-microbe nutrient competition (Grandy et al., 2012; Bardgett et al., 2014). These relationships are further complicated by the changes of soil physical and chemical properties, such as soil moisture content, temperature, and soil structure, that are caused by irrigation techniques and seasonal variations (Barakat et al., 2016).

Frequent and extended droughts have led to a significant shift in irrigation strategies, highlighting the need to better understand root and rhizosphere responses to water and nutrient distribution patterns. In California, which leads the production of organic processing tomato in the United States, the majority of organic processing tomato land area has converted to subsurface drip irrigation (SDI) to increase water use efficiency and decrease weed pressure (Ayars et al., 1999; Sutton et al., 2006; Klonsky, 2010). However, organic growers have experienced mixed results, as SDI often causes low yields, especially in the years following the irrigation method conversion (Taylor and Zilberman, 2017; Schmidt et al., 2018). SDI involves low water inputs delivered in frequent and targeted irrigations, resulting in more localized and constant wetting patterns in the root zone than traditional furrow irrigation (FI), which experience intense wet-dry cycles (Ayars et al., 2015). The consistently wetting (SDI) and wet-dry cycling (FI) patterns have created different spatial distributions in terms of soil moisture, nutrient, and microbial communities in the soil profile (Griffin, 2018; Schmidt et al., 2018). However, understanding remains limited as to how the interplay between water and nutrient patterns under two irrigation strategies influences tomato root developmental patterns and rhizosphere processes that determine plant nutrient uptake and productivity.

To address this knowledge gap, we conducted a field experiment in an organically managed tomato system to compare how SDI and FI impact root distribution, morphological traits, and rhizosphere processes including microbial C-, N-, and P-cycling enzyme activities, the abundance of microbial N-cycling genes, and the extent of mycorrhizal root colonization. We hypothesized that due to more constant and targeted water delivery and localized nutrient placement in SDI than FI: (1) plants grown with SDI will have roots concentrated in patches around the drip line, and plants grown with FI will have relatively diffused root distribution; (2) microbial activities involved in C and nutrient cycling will be greater in the rhizosphere of SDI managed plants than the FI; and (3) plants will have a lower dependence on mycorrhizal colonization in SDI compared to FI.

## Materials and Methods

### Field Site and Experimental Design

The field site was located at the Century Experiment at Russell Ranch Sustainable Agricultural Research Facility (part of the University of California, Davis Agricultural Sustainability Institute) in Winters, California (38.54′N, 121.87′W) (Wolf et al., 2018). Starting in 1994, the Century Experiment was initiated with 11 cropping systems across 72 0.4-ha plots arranged as a randomized complete block design. The climate is the Mediterranean, characterized by mild and rainy winters and hot dry summers. The experimental plots span two soil types: Rincon silty clay loam (fine, montmorillonitic, thermic Mollic Haploxeralfs) and Yolo silt loam (fine-silty, mixed nonacid, thermic Typic Xerorthents) (Wolf et al., 2018).

Since 2015, the comparison of two irrigation strategies (SDI and FI) was initiated in a 2-year certified organic tomato (*Solanum lycopersicum* L.) and maize (*Zea mays* L.) rotation system. The comparison was established by splitting the experimental plot into two sides, with SDI treatment assigned to one side and FI to the other side. Our experiment took place on three replicated plots in the processing tomato phase of the organic tomato-maize rotation in 2017 (i.e., 2 years after the initiation of irrigation comparison) (Schmidt et al., 2018). Processing tomato (variety: Heinz 8504) was transplanted as a single row on a 150 cm wide raised bed, and the planting density was 21,000 plants ha^–1^.

SDI was implemented through a single drip line buried at the center of the bed 25∼30 cm below the soil surface. FI was applied through surface flood irrigation, with alternate irrigation of the two furrows between each bed during the growing season. FI received 11,222 m^3^ ha^–1^ and SDI received 4,839 m^3^ ha^–1^ of irrigation during the 2017 growing season. The weekly irrigation schedule was derived from the irrigation recommendation of Tule (Tule Technologies, United States), which measures actual evapotranspiration (ET) of a field using the Surface Renewal method through *in situ* sensors (Shapland et al., 2014). Tule provides weekly irrigation recommendations based on previous week’s actual ET, plant responses, forecasted atmospheric demand, and the estimation of water demand depending on production goals. The frequency and volume of each irrigation events for FI and SDI was recorded using flow meters over the growing season (Supplementary Figure S1).

**FIGURE 1:**
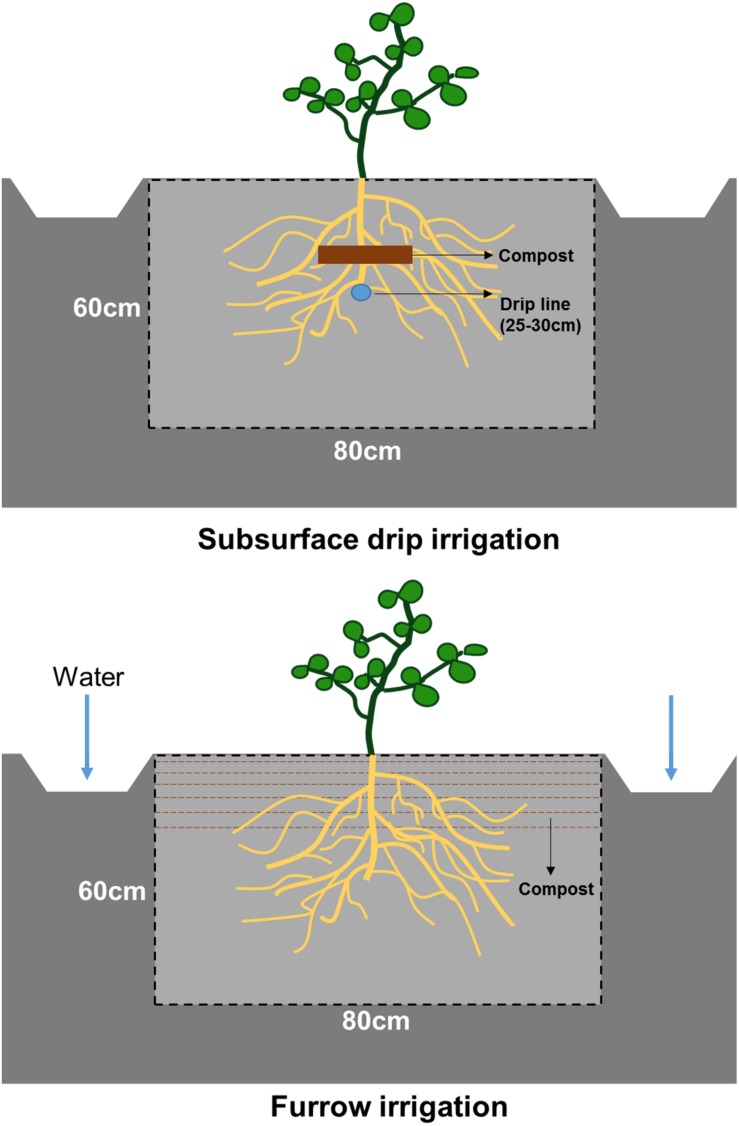
Diagram of irrigation method, fertilizer application, and soil profile surface in subsurface drip and furrow irrigation systems.

Composted poultry manure (9 Mg ha^–1^) contained approximately 20.0% C, 2.0% N, and 1.4% P was applied prior to tomato planting. Both irrigation treatments received the same amount of composted manure but were applied differently: mixing with the top 20 cm of soils in the FI system and banding compost on top of the drip line in the SDI system (Figure 1). Different application methods of composted manure are part of the long-term design of this experiment and implemented to maximize the overlap between fertilizer distribution and the specific moisture pattern of the two irrigation strategies. Winter cover crop was seeded with a mix of legumes and grass, composing of 90.0 kg ha^–1^ bell bean (*Vicia faba* L.), 22.5 kg ha^–1^ lana vetch (*Vicia villosa* Roth), and 28.0 kg ha^–1^ oats (*Avena sativa* L.). Winter cover crop was terminated and incorporated into the soil before compost application and planting.

### Root Distribution and Analysis

Tomato root distribution was characterized on fully developed root systems using detailed field soil profiles and digital analysis taken in both treatments 105 days after transplanting (i.e., 3 days after the irrigation ceased and 2 weeks prior to tomato harvest). To observe root distribution, we dug the soil trench manually using shovels and mattocks on a randomly selected crop row in each treatment. We smoothed the soil profile surface using a sharpshooter spade. The observation trench was 90 cm (length) ^∗^ 80 cm (width) ^∗^ 60 cm (depth) (Figure 1). Root distribution was recorded on the soil profile surface perpendicular to the plant row and tangential to the plant stem. A marked 80 cm ^∗^ 60 cm polyvinyl chloride (PVC) frame was used to define the measurement area and calibrate digital images.

Images of root distribution were taken at 90 cm from the soil profile surface and digitally processed to extract root distribution and morphological trait data. A tent was used to avoid shading effects on root distribution measurements. The smartphone application CamScanner (IntSig Information Co., Ltd, China, version 5.1.2) was used to enhance the contrast and correct deformation. This method was previously tested and shown to provide high-quality images suited for root traits analysis (Mohamed et al., 2017). Processed images were converted to 8-bit gray images using the Fiji platform (Schindelin et al., 2012) and imported into WinRHIZO software (Regent Instruments, Canada) for root analysis. Each image was divided into 48 10-cm^2^ quadrats that were analyzed separately using the WinRHIZO software. Total root length and root diameters were obtained from each quadrat. Root distribution was calculated as the percentage of root length contained in each quadrat relative to the total root length.

### Rhizosphere Soil Sampling and Analysis

Rhizosphere soil was collected and analyzed for microbial extracellular enzyme activity and the abundance of microbial N-cycling genes. Rhizosphere soil samples were collected by brushing adhering soils from root surfaces located in the top 30 cm of the soil profile. Two subsamples were collected for DNA extraction and soil enzyme activity analysis. All soil samples were stored in sterilized Falcon tubes at −20°C until further analyses.

The potential activities of seven extracellular enzymes involved in C cycling (AG: α-glucosidase, BG: β-glucosidase, XYL: β-xylosidase, and CB: β-D-cellobiosidase), N cycling (NAG: *N*-acetyl-glucosaminidase, and LAP: leucine-amino-peptidase), and P cycling (PHOS: acid phosphatase) were measured in rhizosphere soils using the 96-well plate fluorometric method (Bell et al., 2013). Briefly, a 2.75 g soil sample was thoroughly mixed with 91 ml of 50 mM sodium acetate buffer in a blender. The pH of the buffer was adjusted to the average pH of the soil samples (pH = 7.6). The soil slurry was then mixed on a stir plate as 800 μl were transferred to deep 96-well plates. Substrate concentrations and incubation time was determined based on prior tests in order to capture the maximum potential enzyme activity (V_*max*_). We used 600 μM fluorescently labeled substrates for all enzymes assayed except LAP, where we used 375 μM. We pipetted 200 μl of each substrate into the sample assay wells and incubated for 3 h at 25°C. For each sample, we also prepared standard curves using 4-methylumbelliferone or 7-amino-4-methylcoumarin (used for LAP only). After incubation, assay plates were centrifuged for 3 min at 1500 rpm, and 250 μl of supernatant from each well was transferred into black 96-well plates. Substrate fluorescence was measured on a Tecan Infinite M200 (Tecan Trading AG, Switzerland) microplate reader at wavelengths 365 nm (excitation) and 450 nm (emission). The enzyme activity of each rhizosphere soil sample was calculated based on the soil dry weight and incubation time (unit: nmol g^–1^ h^–1^).

To quantify the abundance of microbial genes involved in N-cycling processes, DNA was extracted from a 0.25 g subsample of each rhizosphere soil sample using the DNeasy PowerSoil DNA isolation kits (Qiagen, Germany) according to the manufacturer’s protocol. The N-cycling functional genes involved in nitrification (archaeal *amoA* and bacterial *amoA*), denitrification (*nosZ*), N-fixation (*nifH*), and dissimilatory nitrate reduction to ammonium (DNRA) (*nrfA*) were quantified by quantitative PCR (qPCR) using the microfluidic Access Array (Fluidigm Corporation, United States) at the Roy J. Carver Biotechnology Center (University of Illinois at Urbana-Champaign, IL, United States). Genes were amplified using the primers described in Supplementary Table S1. A specific target amplification (STA) was used to increase the amount of template for each target gene prior to Fluidigm qPCR. The STA pre-amplification reaction was performed in 5 μl reaction mixtures containing 2 × Taqman PreAmp Master Mix (Applied Biosystems, United States), 0.5 μM of each primer, and 1.25 μl of the DNA template extracted from rhizosphere soil. The STA reaction was performed on an MJ Research Tetrad thermal cycler with the following cycling program: 95°C for 10 min followed by 14 cycles of 95°C for 15 s and 58°C for 4 min. Standards of each gene were derived from soil microbial communities, quantified, mixed. A fivefold dilution series from 1 × 10^5^ to 3.2 × 10^1^ copies μl^–1^ was subjected to the STA pre-amplification reaction along with the soil DNA to provide standard curves for Fluidigm qPCR. The STA products were treated by exonuclease to remove excessive primers. STA products were amplified on the Fluidigm Biomark HD Real-Time PCR. All the samples and standards were analyzed in 12 technical replicates. The C_*T*_ values (cycle threshold) and copy numbers for each gene were determined using Fluidigm Real-Time PCR Analysis software version 4.1.3. Mean values and standard errors were expressed as the number of copies per ng of genomic DNA (quantified by Qubit, Invitrogen, United States) from technical replicates with quality scores of at least 0.65.

### Root Sampling and Mycorrhizal Colonization

Two tomato plants were uprooted from the same row where the soil observation profile was excavated, and roots were thoroughly washed to remove soil particles. Fine lateral roots were randomly collected from the whole root system and stored in 50% ethanol at room temperature for further measurement. For colonization quantification, roots were cleared in hot 10% potassium hydroxide solution for 5 min, acidified in 2% hydrochloric acid for 15 min, and stained with preheated 0.05% direct blue dissolved in the 1:1:1 (v/v/v) mixture of water, glycerin, and lactic acid (INVAM, 2017). Mycorrhizal colonization rate was quantified using the grid-intersect method based on at least 100 intersects per sample (Giovannetti and Mosse, 1980). Percentage of root length colonized was determined as the proportion of root intersects containing any mycorrhizal structure (i.e., arbuscules, vesicles, and hyphae).

### Soil Sampling and Analysis

Soil gravimetric water content was measured on ∼80 g soil samples taken from the soil observation profile at three distances across the bed (at the center and 27 cm left and right from the bed center) and at three depths (10, 30, and 50 cm) (Supplementary Figure S2). The distribution of soil nutrients (i.e., N, P, K) was measured at a higher spatial resolution: 10, 25, and 45 cm from the center of the bed and at 15, 30, and 45 cm depth using a 5 cm diameter soil core. Soil samples were kept in a cooler with ice during sampling and then stored at 4°C until extraction or air-drying. Soil ammonium-N (NH_4_^+^-N) and nitrate-N (NO_3_^–^-N) were extracted from 20 g of field moist soil (within 7 days of sampling) with 100 ml of 0.5 M potassium sulfate (K_2_SO_4_), shaken on a reciprocal shaker for 1 h, then filtered through Fisherbrand Q5 filter paper. Extracts were frozen at −20°C until analysis. Soil ammonium-N (NH_4_^+^-N) and nitrate-N (NO_3_^–^-N) concentrations were determined colorimetrically with salicylate-hypochlorite and vanadium (III) chloride reduction methods, respectively (Verdouw et al., 1978; Doane and Horwáth, 2003). Reactions were scaled down to be done in 96-well microplates (385 μL capacity; Sarstedt, Inc., Newton, NC, United States), and absorbance values were measured on a Tecan GENios microplate reader at 650 nm (ammonium) and 540 nm (nitrate). Soil available P and exchangeable K were measured by the UC Davis Analytical Laboratory on air-dried soil samples collected on 15 June 2017 (during peak nutrient uptake). The gravimetric water content of each sample was used to calculate inorganic N concentrations on an oven-dry weight basis. Soil P availability was determined by the sodium bicarbonate (NaHCO_3_) extraction according to the Olsen method (Olsen, 1954), and soil exchangeable K was determined by the ammonium acetate extraction followed by emission spectrometry (Knudsen et al., 1982).

### Aboveground Biomass, Nutrient Content, and Yields

Tomato plants were hand-harvested in both irrigation treatments from two randomly selected 2-m long sections (6–7 plants) per replicate. The aboveground biomass was separated into marketable fruits, green fruits, and stem and leaf tissues, and fresh weight was recorded for each component. Tomato yields were calculated based on the fresh weight of marketable fruits (i.e., the red and orange tomato fruits that can be used for canning). Irrigation water productivity (Mg m^–3^) was calculated by dividing the marketable yields by the volume of irrigation water applied. The aboveground plant tissues were oven-dried (60°C) for 3 days and ground. Total plant N was determined by the combustion method (Sweeney, 1989), and total P and potassium (K) were measured using Inductively Coupled Plasma Spectroscopy at the Ward Laboratories, Inc., NE, United States.

### Statistical Analysis

Analysis of variance (ANOVA) with treatment as a fixed effect and block as a random effect was used to test the effect of irrigation method on tomato yield components, total root length, average root diameter, rhizosphere soil enzyme activity, the abundance of N-cycling genes, and mycorrhizal colonization rate. For the percentage of root length at different positions, ANOVA was conducted for each position separately using treatment as the fixed effect and block as a random effect. For the soil nutrient analysis, ANOVA was conducted using treatment, distance from bed center, depth, and their interactions as fixed effects while block as a random effect. The assumptions of normal distribution and homogeneity of variance of the residuals were verified with the Shapiro–Wilk normality test and Levene’s test, and log-transformation was applied as needed. All statistical analyses were conducted in R 3.4.1 (R Core Team, 2018).

## Results

### Root Distribution

**FIGURE 2:**
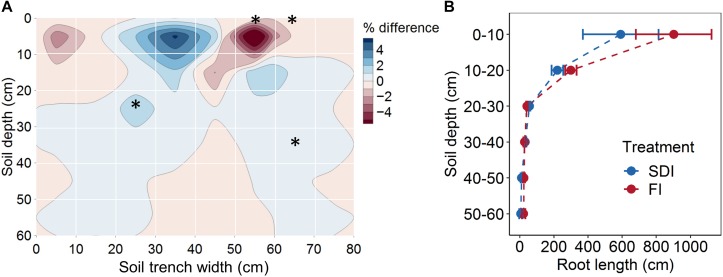
**(A)** Root distribution by position and **(B)** root length at different soil depths of tomato plants in subsurface drip irrigation (SDI) and furrow irrigation (FI) systems. Root distribution in **(A)** was calculated as the difference in the percentage of root length contained in each quadrat between two irrigation treatments. Blue shades represent a higher percentage in SDI than FI, and red shades represent a lower percentage in SDI than FI. Asterisks represent significant differences (*p* < 0.05) between two irrigation systems. Error bars represent the standard error of the mean.

**FIGURE 3:**
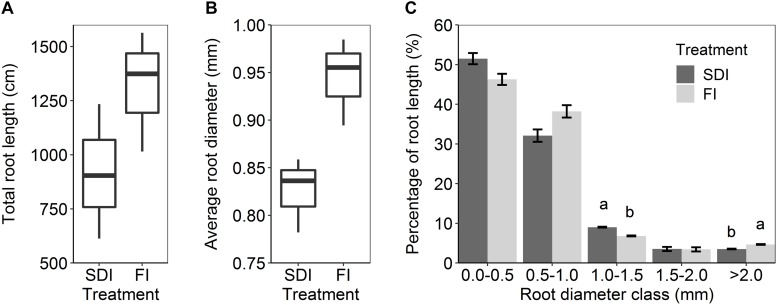
**(A)** Total root length, **(B)** average root diameter, and **(C)** the percentage of root length in different diameter classes in subsurface drip irrigation (SDI) and furrow irrigation (FI) systems. Letters represent significant differences (*p* < 0.05) between two irrigation treatments. Error bars represent the standard error of the mean.

Subsurface drip irrigation with banded compost resulted in a more concentrated root distribution pattern than the furrow irrigation with compost incorporated into topsoil. SDI showed a higher percentage of root length (1.52%, *F*_1_,_2_ = 18.107, *p* = 0.051) at the depth of 20–30 cm, 10–20 cm away from the drip line than FI (Figure 2A). Similarly, a higher percentage of root length (0.24%, *F*_1_,_2_ = 33.239, *p* = 0.029) was found at the depth of 30–40 cm, 20–30 cm away from the drip line in SDI than in the FI system (Figure 2A). Conversely, FI led to higher root distribution toward the edge of the bed and at greater depth relative to SDI. A greater percentage of roots (7.72%, *F*_1_,_2_ = 33.213, *p* = 0.029) was found in the top 10 cm of soil and at 10–20 cm and 20–30 cm from the bed center (2.49%, *F*_1_,_2_ = 78.640, *p* = 0.013) in the FI than the SDI system (Figure 2A). Shifts in the distribution of root length were mostly driven by fine roots in the 0.0–0.5 mm diameter class (Supplementary Figure S3). Regardless of irrigation method, ∼94% of tomato roots were concentrated in the top 30 cm of the soil profile, and root length decreased rapidly with increasing soil depth (Figure 2B). SDI resulted in a substantial decrease in total root length and shift toward finer roots compared to FI, as shown by a 30.4% lower total root length (*F*_1_,_2_ = 1.391, *p* = 0.360) and a 12.6% decrease in average root diameter in SDI than the FI system (*F*_1_,_2_ = 12.759, *p* = 0.070) (Figures 3A,B). Compared to FI, SDI led to a higher percentage of root length in the 1.0–1.5 mm root diameter class, but a lower percentage of coarser roots (>2.0 mm class) (Figures 3B,C).

### Rhizosphere Nutrient Cycling and Mycorrhizal Colonization

There was a trend of lower activity of organic C-cycling enzymes in the rhizosphere of SDI plants relative to FI. The activity of AG in the rhizosphere of SDI plants was 23.2% lower than FI (Table 1 and Supplementary Figure S4). The activity of CB, XYL, and BG was less affected by irrigation systems but also showed trends of decreased activities in the SDI system than in the FI system (Table 1 and Supplementary Figure S4). Activities of N- and P-cycling enzymes remained unchanged between the two irrigation systems (Table 1 and Supplementary Figure S4). The abundance of microbial functional genes involved in different steps of the N-cycling process was also not statistically different between the two irrigation treatments (Table 2 and Supplementary Figure S4). However, it is worth noting that there is a trend of higher N cycling genes in the rhizosphere of SDI plants than FI. In particular, the abundance of the bacterial nitrification gene *amoA*, the most abundant gene involved in N cycling, was on average 60.0% higher in the rhizosphere of SDI irrigated tomatoes than in FI (Table 2 and Supplementary Figure S4). Mycorrhizal colonization rates were on average 13.6 and 10.8% for SDI and FI irrigated plants, respectively.

**TABLE 1:** The activity of soil extracellular enzymes involved in carbon (C), nitrogen (N), and phosphorus (P) cycling in the rhizosphere of tomato plants under furrow irrigation (FI) and subsurface drip irrigation (SDI).

Group	Target enzyme	Enzyme activity (nmol substrate g^–1^ soil h^–1^)		*P*-value
		
		SDI	FI	
Carbon cycling enzymes	α-glucosidase	7.9 (1.1)	10.3 (0.7)	0.054
	β-glucosidase	78.3 (3.0)	82.7 (4.6)	0.546
	β-xylosidase	20.0 (3.0)	23.6 (2.5)	0.104
	β-D-cellobiosidase	16.7 (1.6)	19.9 (2.2)	0.369
Nitrogen cycling enzymes	*N*-acetyl-glucosaminidase	37.9 (0.8)	39.2 (3.1)	0.728
	Leucine-amino-peptidase	40.6 (4.8)	44.2 (6.7)	0.582
Phosphorus cycling enzymes	Acid phosphatase	332.6 (29.6)	323.9 (29.1)	0.599

*Numbers in parentheses represent standard error of the mean (*n* = 3).*

**TABLE 2:** The abundance of microbial genes involved in nitrogen (N) cycling in the rhizosphere of tomato plants under furrow irrigation (FI) and subsurface drip irrigation (SDI).

Group	Target gene	Gene abundance (copies ng^–1^ genomic DNA)		*P*-value
		
		SDI	FI	
Nitrification	Bacterial *amoA*	11,426.8(2944.1)	7,139.6(1582.1)	0.659
	Archaeal *amoA*	750.1 (185.0)	602.7 (109.6)	0.437
Denitrification	*nosZ*	461.4 (114.2)	395.2 (39.6)	0.672
N-fixation	*nifH*	262.8 (22.4)	238.7 (40.2)	0.638
Dissimilatory nitrate reduction to ammonium	*nrfA*	3,739.5(425.1)	3,787.5(422.0)	0.918

*Numbers in parentheses represent standard error of the mean (*n* = 3).*

### Soil Nutrient Distribution

**FIGURE 4:**
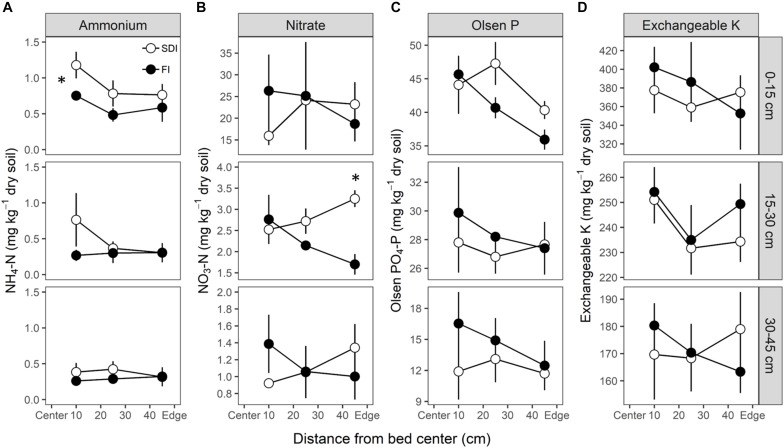
The distribution of soil available **(A,B)** nitrogen (N), **(C)** phosphorus (P), and **(D)** potassium (K) in subsurface drip irrigation (SDI) and furrow irrigation (FI) systems. Ammonium-N and nitrate-N were collected in August, and Olsen-P and exchangeable K were obtained in June. An asterisk on the left side of the plot represents a significant treatment difference (*p* < 0.05) at all distances across the bed, while an asterisk above one point represents a significant treatment difference at one distance.

The distribution of soil available N (i.e., ammonium-N and nitrate-N), Olsen-P, exchangeable K varied depending on soil depth and distances to the bed center (Figures 4A–D). Soil ammonium-N was higher in SDI in the topsoil (0–15 cm) (*p* = 0.026) at the time of harvest relative to the FI system (Figure 4A). At 10 cm from the bed center, where the majority of roots are located, soil nitrate-N, Olsen-P, and exchangeable K were not different between the two irrigation treatments but tended to be lower in the SDI than the FI system at all depths (Figures 4B–D). Soil nitrate-N was substantially and consistently higher than the ammonium-N throughout the soil profile. Nitrate-N levels at the 15–30 cm depth showed a treatment^∗^distance interaction (*p* = 0.008), with higher nitrate-N at the edge of the bed in the SDI compared to the FI (*p* = 0.015) (Figure 4B).

### Tomato Yield, Irrigation Water Productivity, and Plant Nutrient Content

**FIGURE 5:**
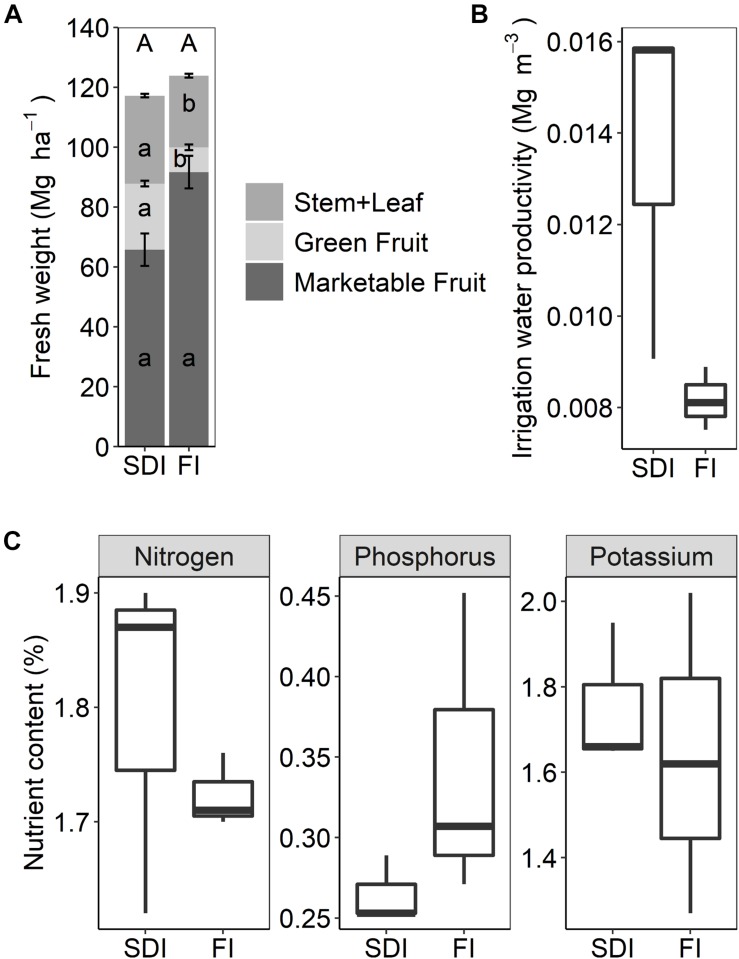
**(A)** Marketable yields and aboveground biomass of processing tomato, **(B)** boxplots of irrigation water productivity, and **(C)** boxplots of nitrogen, phosphorus, and potassium content in aboveground plant tissues in subsurface drip irrigation (SDI) and furrow irrigation (FI) systems. Letters represent significant differences (*p* < 0.05) between two irrigation treatments. Error bars represent the standard error of the mean.

Two irrigation systems (SDI vs. FI) significantly affected biomass allocation to different aboveground plant tissues. Compared to FI, marketable tomato yield decreased by 28.3% in the SDI system (*F*_1_,_2_ = 11.255, *p* = 0.076), while the biomass of non-marketable fruits (*F*_1_,_2_ = 110.370, *p* = 0.009) and stems and leaves increased (*F*_1_,_2_ = 40.600, *p* = 0.024) (Figure 5A). SDI substantially reduced the amount of irrigation water applied, leading to 66.4% increase in irrigation water productivity compared to the FI system (*F*_1_,_2_ = 7.729, *p* = 0.109) (Figure 5B). Plant N, P, and K contents were not different between SDI and FI systems (Figure 5C).

## Discussion

Innovations in irrigation technology usually focus on optimizing timing, wetting patterns, and intensity of irrigation to increase resource use efficiency with the preset assumption of crop root distribution. However, the plasticity of root systems and the interactions between roots and rhizosphere microbes that mediate plant nutrient uptake and soil nutrient cycling have been largely neglected (Raine et al., 2007). Our study tackles this knowledge gap by assessing responses of root traits and rhizosphere processes to moisture and nutrient patterns determined by irrigation strategies in an organic system and provides insights into their relationships with plant nutrient uptake, yield, and soil nutrient cycling. As hypothesized, plant roots showed high plasticity in adapting to resource heterogeneity induced by irrigation, with the development of a finer root system and a higher concentration of roots around the drip line in SDI relative to the FI system. Rhizosphere processes also showed patterns of coordinated adaption to shifts in soil moisture and nutrient patterns associated with SDI and FI. These included lower activities of soil organic C degrading enzymes, but trends of increased root mycorrhizal colonization and microbial N cycling genes in the rhizosphere of SDI tomato plants relative to FI. In addition, SDI resulted in excessive vegetative growth and lower tomato marketable yields compared to FI. Our results suggest that, despite regulation of plant-microbe interactions and root morphological traits to exploit the resource heterogeneity induced by irrigation, mismatches remained between plant needs, resource availability, and microbial C/N transformation processes.

We observed that root distributional and morphological traits adapted to different moisture and nutrient patterns induced by irrigation and its associated fertilization methods. SDI and FI create distinct distributions of water and nutrients (Figures 4A–D and Supplementary Figure S2), which require substantially different root morphological and physiological traits for resource exploitation. In our organic SDI system, the localized placement of organic fertilizer overlaps with constant soil moisture likely creating a resource-rich hotspot around the drip line (Figure 1 and Supplementary Figure S2). This resource pattern requires a root ideotype with prolific fine lateral roots and high uptake rate to efficiently utilize soil available nutrients in the hotspot (Schmidt and Gaudin, 2017). The low root-zone nitrate-N and high plant N concentrations observed in SDI plants, despite their relatively small root system, provide evidence for efficient exploitation of the resource-rich hotspot via localized root proliferation. In contrast, under FI, the organic N source was mixed throughout the upper 20 cm of soil and was subjected to more intensive wet-dry cycles (Figure 1). This resource pattern requires roots to explore and forage in a larger soil area and remain relatively unresponsive to water fluctuations (Hodge, 2009; Schmidt and Gaudin, 2017). The development of a thicker and diffuse root system in the FI system enables plants to explore larger soil area and adapt to intensive dry-wet cycles (Jackson and Bloom, 1990; Zotarelli et al., 2009a). Our results are consistent with previous studies in conventional systems, where up to 96% of tomato roots were found in the top 0–40 cm of the soil around the drip line (do Rosaìrio et al., 1996; Machado et al., 2003; Zotarelli et al., 2009b).

Our soil trench method with root image analysis also showed results consistent with different root quantification methods such as *in situ* root mapping (Araujo et al., 1995), wet sieving roots out of soil cores (Hodgson et al., 1990), or installing minirhizotrons in the root zone (Machado et al., 2003). These root architectural and morphological traits provide evidence that roots have a high potential for coordinated morphological adaptation to resource heterogeneity under different irrigation strategies. It is also worth mentioning that we applied organic fertilizers accordingly in two systems to reflect growers’ practices and to optimize the overlap between fertilizer and moisture distributions (Figure 1). Therefore, the observed changes in root distribution were a function of irrigation methodology and its associated nutrient application method.

The overall abundance of microbial functional genes involved in key N-cycling processes tended to increase in the rhizosphere of SDI as compared to FI (Supplementary Figure S4), especially the ammonium-oxidizing microbes involved in nitrification. A previous greenhouse study based on amplicon sequencing also found a similar response (Wang et al., 2018). The abundance of ammonium-oxidizing microbes often increase in constantly moist soils, but decrease in saturated soils due to reduced oxygen availability and in dry soils due to reduced diffusion of substrate supply and cell dehydration (Stark and Firestone, 1995). In addition, high ammonium concentrations in the soil can increase the population size of ammonium-oxidizing microbes (Okano et al., 2004). Therefore, the constant wetting due to drip irrigation and high ammonium concentration due to banded compost in the root zone in SDI (Figure 4A and Supplementary Figure S2) may lead to a higher abundance of nitrifiers relative to FI. Increased abundance of nitrifiers will lead to faster conversion of ammonium to nitrate. When nitrogen is in the form of highly soluble and mobile nitrate, there is increased diffusion of N in the root zone (Jackson et al., 2008) but may also be greater potential for N losses by leaching and denitrification (Coskun et al., 2017). The localized proliferation of roots in SDI may facilitate more efficient uptake of soil available N and mitigate N losses through leaching from the root zone (Zotarelli et al., 2009a). On the other hand, the diffuse and thick roots developed in the FI system may be less efficient at nutrient uptake (Jackson and Bloom, 1990). The relatively low plant N concentration and significant decreases in soil nitrate-N toward the bed edge in FI may reflect potential losses of nitrate-N when excessive water increases the leaching of nitrate below the root zone (Bowles et al., 2018). Previous studies have reported higher N use efficiency in SDI relative to FI and had mainly attributed the benefit to precise placement of water and fertilizers in the root zone (Ayars et al., 2015). Here, we suggest that coordinated changes in root morphological traits and rhizosphere N-cycling microbial communities may also play a role in increasing N use efficiency in SDI as compared to FI. Although the relationship between gene abundances and corresponding biogeochemical process rates may be complicated by various environmental, biological, and methodological factors, quantifying the abundance of N-cycling genes is still a valuable approach to understand the dynamics of microbial processes responsible for N transformations (Rocca et al., 2015; Ouyang et al., 2018; Ma et al., 2019). Future breeding efforts toward enhancing nutrient transporter affinity, root system plasticity, and root exudate regulation of N-cycling rhizosphere microbial communities may provide opportunities for promoting plant N uptake and N retention in agricultural systems (Rengel and Marschner, 2005; Jackson et al., 2012).

Plants under SDI showed a trend of higher AM fungal colonization than under FI (Supplementary Figure S4), indicating potential increased investment in mycorrhizal associations to acquire soil nutrients. Plants often show high dependence on AM fungi to enhance P uptake in low P soils, but reduced fungal colonization under high soil P conditions due to decreased benefits of resource acquisition and high C cost to host plants (Li et al., 2016). The trend of lower P content in plant tissues (Figure 5C) together with relatively lower root-zone P availability (Figure 4C) may lead to the increased dependence of plants on AM fungi in SDI relative to FI, where frequent wet-dry cycles likely increased soil P availability due to mineralization and aggregate disruption (Liu et al., 2017; Wang et al., 2017). Enhanced AM fungal colonization of organic tomatoes has been associated with high marketable yields, increased plant N and P concentrations, and improved water uptake capacity under SDI (Bowles et al., 2016). Given the benefits of AM fungi, promoting their contributions through management practices or increasing establishment of target AM fungi through inoculation may provide promising opportunities to improve yields and nutrient status of processing tomatoes in organic systems under SDI (Cely et al., 2016).

Interestingly, soil C-cycling extracellular enzymes showed lower potential activities in the rhizosphere of SDI plants than FI. Lower potential enzyme activity, on one hand, suggests that microbes are less nutrient-limited in the rhizosphere of SDI relative to FI. This is possibly due to a better spatial coincidence of localized organic fertilizer, moisture, and the labile C inputs from plants in SDI than FI (Zhu et al., 2014). In FI, frequent wet-dry cycles can promote the formation of macroaggregates (Schmidt et al., 2018), which may physically protect part of SOM from the microbial attack in the rhizosphere (Denef et al., 2001). On the other hand, lower soil enzyme activity may also result from reduced enzyme production due to a smaller microbial pool in SDI relative to FI. This hypothesis is supported by a previous study where lower microbial biomass was observed across the bed at the top 0–15 cm in SDI than FI (Griffin, 2018). Wet-dry cycles in the FI system may have increased root exudation that fuels microbial growth, leading to increased extracellular enzyme production and enzyme activity (Zhu et al., 2014). The decreased soil macroaggregates and microbial biomass (Griffin, 2018; Schmidt et al., 2018), along with our observation of lower C-cycling enzyme activity suggest the potential for reduction in long-term SOM pools that sustain organic production in SDI than FI. Decreased SOM may also diminish cropping system resilience to frequent and extreme weather events in the face of future climate change, especially in semi-arid areas that tend to have relatively low SOM stocks (Li et al., 2019).

Despite changes in root traits and rhizosphere microbial processes in response to different irrigation and nutrient application methods, we found substantially higher irrigation water productivity but lower marketable tomato yields in SDI than FI. Compared to FI, SDI resulted in 66.4% increase in irrigation water productivity but 28.3% decrease in marketable yields, which is consistent with recent research studies and anecdotal observations from organic growers in California (Taylor and Zilberman, 2017; Schmidt et al., 2018). The high irrigation water productivity in SDI confirmed its benefit in increasing crop water use efficiency, which is increasingly important given the predicted increase of frequent and extensive droughts, especially in California. However, low marketable yields highlight the potential temporal mismatches between soil resource availability and plant demand as a function of changes in root distribution and rhizosphere processes. As shown in our results, the low marketable yields may arise from the shift in biomass allocation to non-yield components and delayed fruit maturation. For example, excessive water and nutrients uptake during the flowering stage can lead to both excessive vegetative growth and a delay in fruit ripening (Scholberg et al., 2000; Steduto et al., 2012). Therefore, future studies are needed to maximize the water-saving benefit of SDI in organic systems and especially reconcile plant-specific responses to resource distribution with soil-specific resource status to avoid potential mismatches of inputs and plant demand. For example, applying regulated deficit irrigation during the growing season based on manipulating plant root-to-shoot signaling may mitigate the delay in fruit ripening, increase fruit quality, and save water in SDI systems (Johnstone et al., 2005; Dodd, 2009).

## Conclusion

Our results suggest that roots and their interactions with rhizosphere microbes exhibit high potential for coordinated adaptation to water and nutrient patterns under different irrigation strategies and showed how they influence plant water use efficiency, yield, and nutrient cycling. Our study was designed to capture fully developed root systems and to evaluate the cumulative effects of nutrient cycling in the rhizosphere. Thus, we did not address the temporal aspects of dynamic root–soil–microbe interactions. A critical next step incorporating analysis of multiple time points during the growing season will enable a full understanding of the complexity of root–soil–microbe interactions and facilitate the design of future irrigation strategies. In addition, future studies in organic systems are needed to enhance root morphological and physiological plasticity, harness beneficial microbes for improved nutrient cycling, and optimize irrigation strategies that maximize the resource-mining potential of roots and rhizosphere processes. These innovations will enhance the ability of agroecosystems to better utilize biological processes rather than costly inorganic fertilizer and irrigation inputs and build up the resilience of agroecosystems in the face of future climate change and resource shortages.

## Data Availability Statement

The datasets generated for this study are available on request to the corresponding author.

## Author Contributions

ML, AG, and KS conceived and designed the experiment. ML, DL, PL, AK, and MM contributed to the sample collection and data analysis. ML wrote the first draft of the manuscript, which was further substantially reviewed by JS. All authors contributed to reviewing and revising of the manuscript.

## Conflict of Interest

The authors declare that the research was conducted in the absence of any commercial or financial relationships that could be construed as a potential conflict of interest.
