# Supplementary material for: Impact of Irrigation Strategies on Tomato Root Distribution and Rhizosphere Processes in an Organic System
Source: Front Plant Sci. 2020 Mar 27;11:360. doi: 10.3389/fpls.2020.00360 (PMC7118217; doi:10.3389/fpls.2020.00360)
Supplement: Supplementary file 1 [file Data_Sheet_1.PDF]

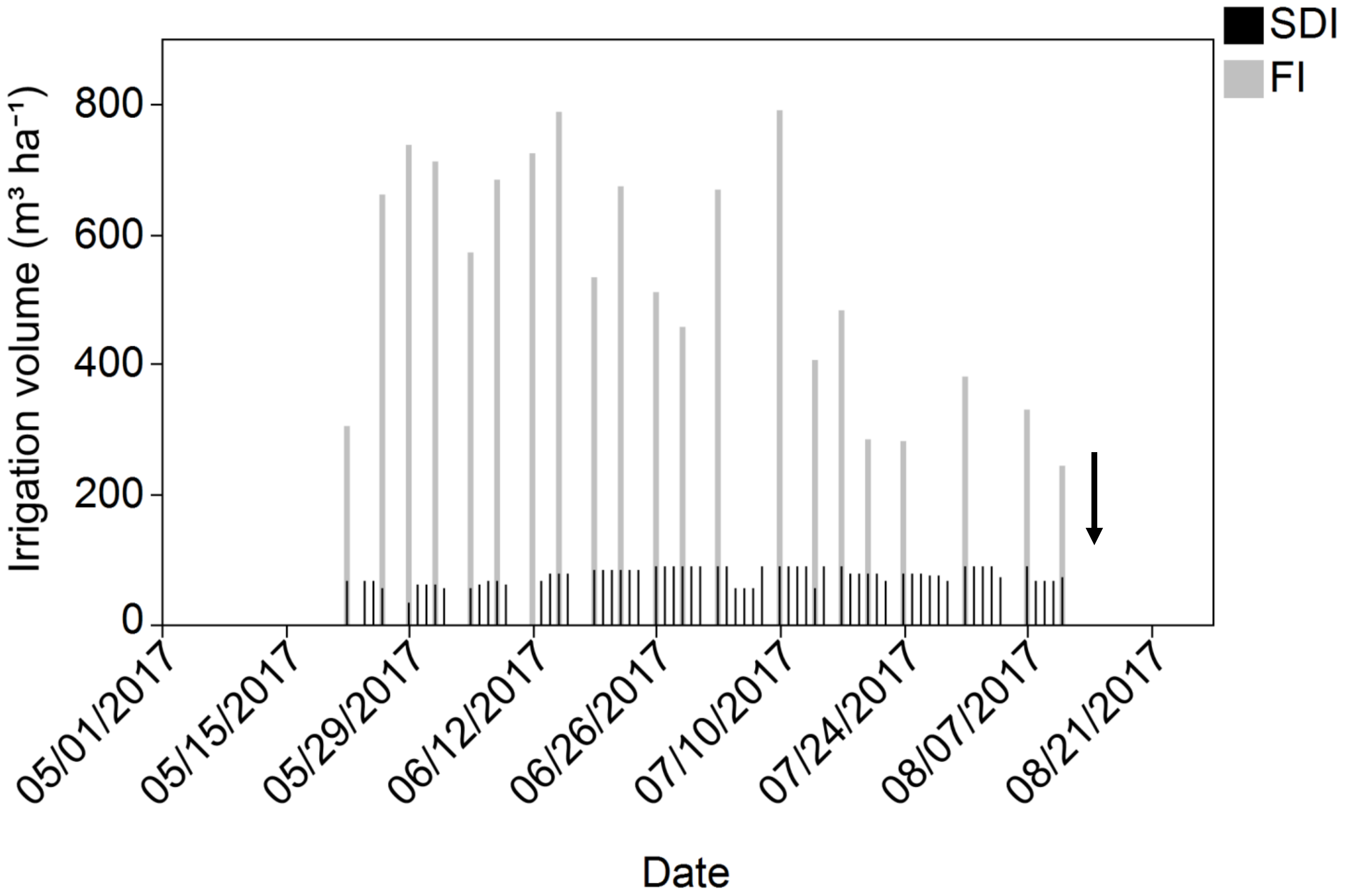

Supplementary Figure S1. Daily irrigation water input of subsurface drip irrigation (SDI) and furrow irrigation (FI) from transplanting (May 1, 2017) to harvest (August 24, 2017) of the 2017 growing season. Arrow represents the sampling date.

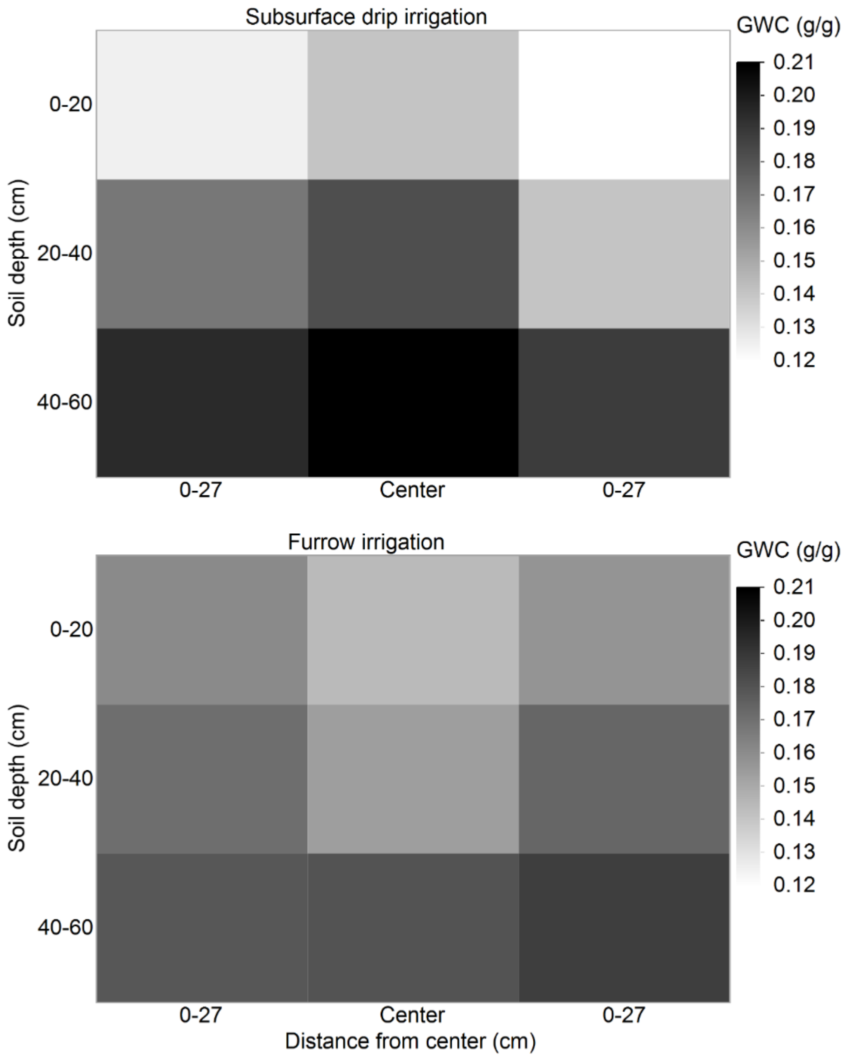

Supplementary Figure S2. The impact of subsurface drip irrigation and furrow irrigation on the distribution of soil gravimetric water content (GWC) at harvest.

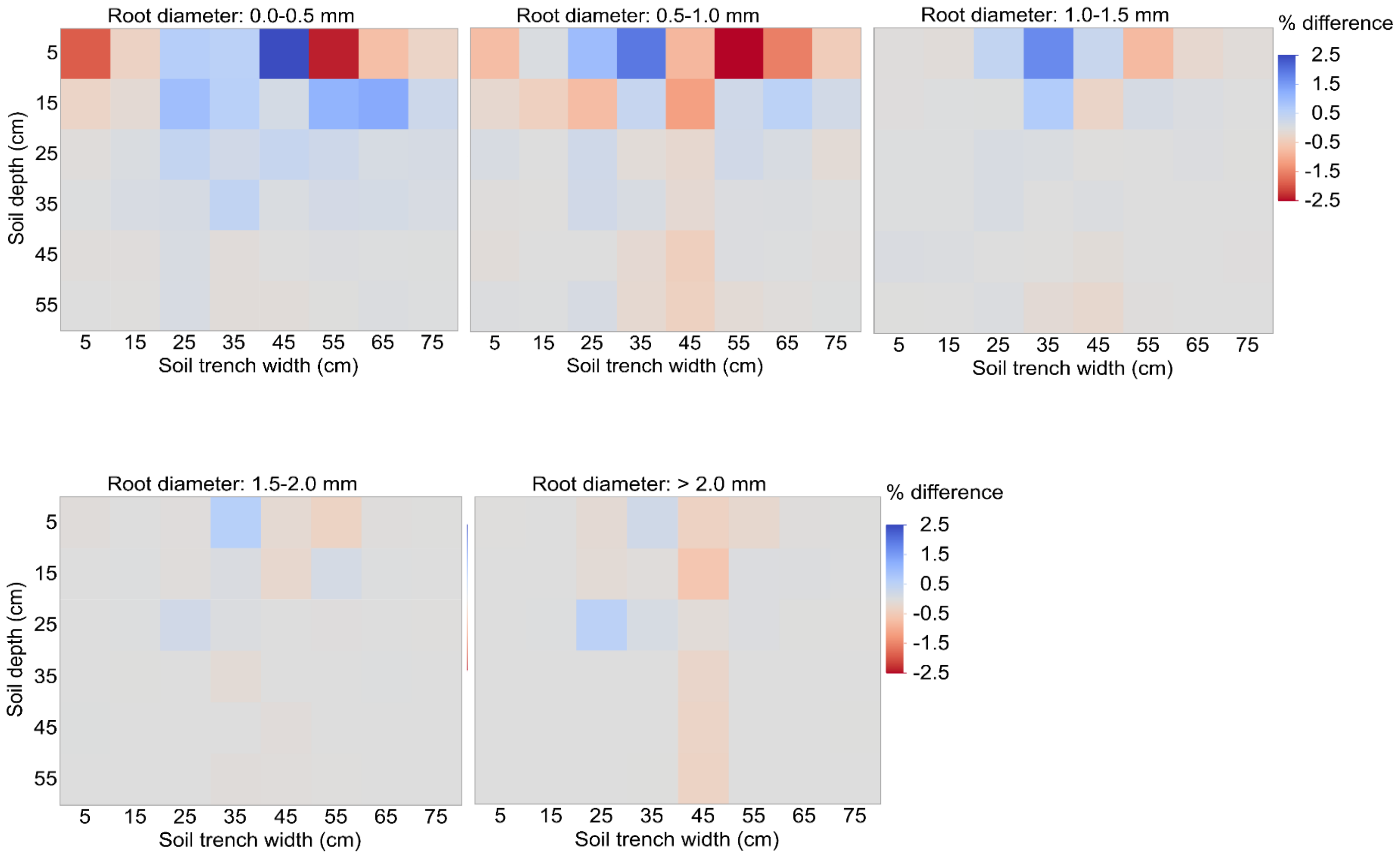

Supplementary Figure S3. Differences of root length distribution in root diameter classes between subsurface drip irrigation (SDI) and furrow irrigation (FI). Shown is the difference (SDI-FI) in percent total root length for each root diameter class. Positive numbers represent increase in SDI compared to FI.

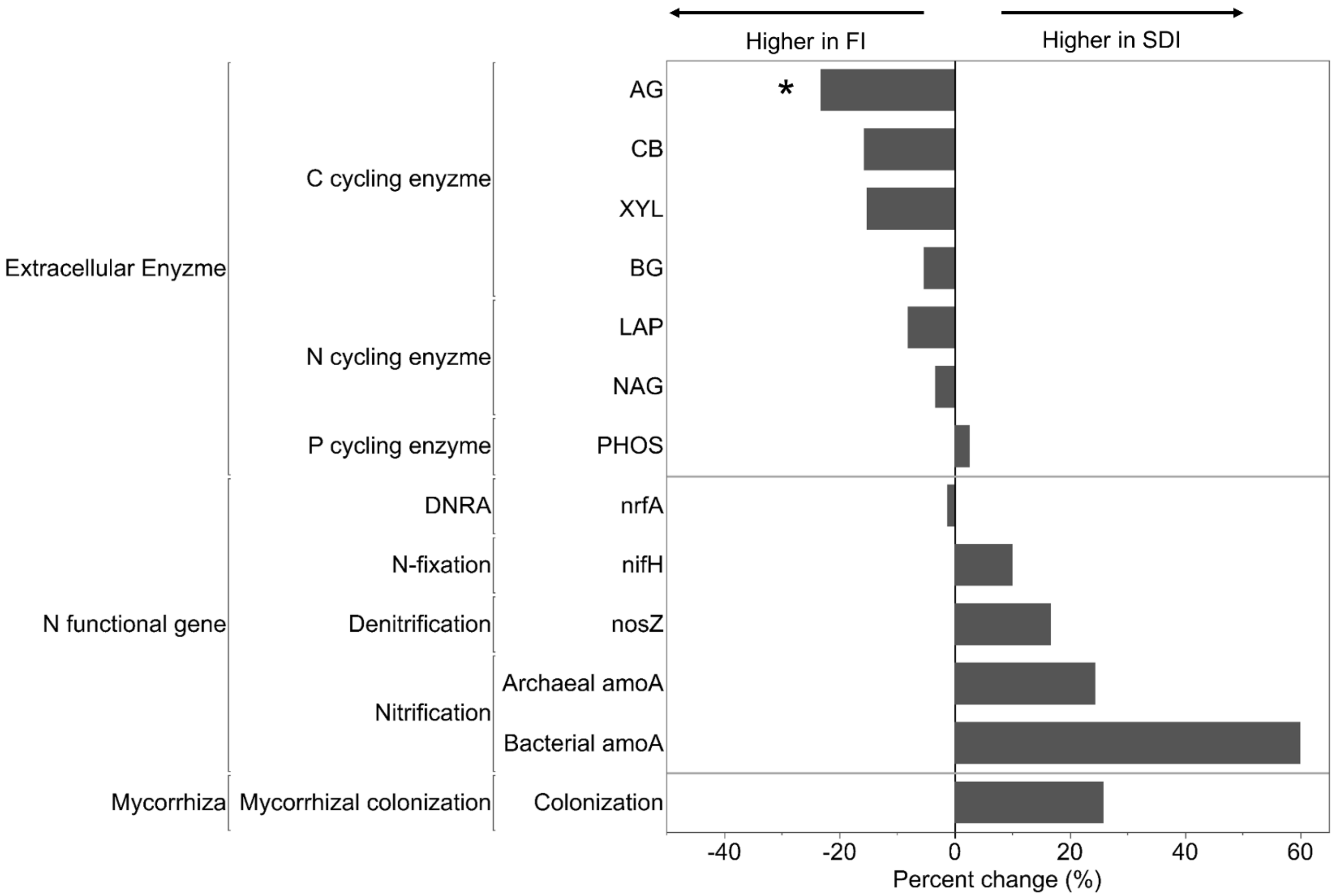

Supplementary Figure 4. The influence of subsurface drip irrigation (SDI) and furrow irrigation (FI) on rhizosphere processes of tomato plants. Bars represent the percentage change in values of SDI compared to FI. Asterisks represent significant differences ($p < 0.05$) between two irrigation treatments. Abbreviations: dissimilatory nitrate reduction to ammonium (DNRA), α-glucosidase (AG), β-glucosidase (BG), β-xylosidase (XYL), β-D-cellobiosidase (CB), N-acetyl-glucosaminidase (NAG), leucine-amino-peptidase (LAP), and acid phosphatase (PHOS).

Supplementary Table S1. Primers used for quantifying the abundance of microbial functional genes involved in N-cycling.

**Gene**	**N-cycling step**	**F primer**	**R primer**	**References**
nifH	N fixation	PolF	PolR	(Poly et al. 2001)
amoA (AOA)	Archaeal nitrification	CreamoA23f	CreamoA616r	(Tourna et al. 2008)
amoA (AOB)	Bacterial nitrification	amoA-1F	amoA-2R	(Leininger et al. 2006)
nrfA	DNRA	nrfAF2aw	nrfAR1	(Welsh et al. 2014)
nosZ	Denitrification	nosZ1F	nosZ1R	(Henry et al. 2006)

References:

Henry S, Bru D, Stres B, et al (2006) Quantitative Detection of the nosZ Gene, Encoding Nitrous Oxide Reductase, and Comparison of the Abundances of 16S rRNA, narG, nirK, and nosZ Genes in Soils. Appl Environ Microbiol 72:5181–5189. doi: 10.1128/AEM.00231-06

Leininger S, Urich T, Schloter M, et al (2006) Archaea predominate among ammonia-oxidizing prokaryotes in soils. Nature 442:806–809. doi: 10.1038/nature04983

Poly F, Monrozier LJ, Bally R (2001) Improvement in the RFLP procedure for studying the diversity of nifH genes in communities of nitrogen fixers in soil. Res Microbiol 152:95–103. doi: 10.1016/S0923-2508(00)01172-4

Tourna M, Freitag TE, Nicol GW, Prosser JI (2008) Growth, activity and temperature responses of ammonia-oxidizing archaea and bacteria in soil microcosms. Environ Microbiol 10:1357–1364. doi: 10.1111/j.1462-2920.2007.01563.x

Welsh A, Chee-Sanford JC, Connor LM, et al (2014) Refined NrfA phylogeny improves PCR-based nrfA gene detection. Appl Environ Microbiol 80:2110–2119. doi: 10.1128/AEM.03443-13
